# Exploring the roles of and interactions among microbes in dry co-digestion of food waste and pig manure using high-throughput 16S rRNA gene amplicon sequencing

**DOI:** 10.1186/s13068-018-1344-0

**Published:** 2019-01-04

**Authors:** Yan Jiang, Conor Dennehy, Peadar G. Lawlor, Zhenhu Hu, Matthew McCabe, Paul Cormican, Xinmin Zhan, Gillian E. Gardiner

**Affiliations:** 1 Civil Engineering, College of Engineering & Informatics, National University of Ireland, Galway, Ireland; 2 Pig Development Department, Animal & Grassland Research and Innovation Centre, Moorepark, Teagasc, Fermoy, Co. Cork, Ireland; 3 School of Civil Engineering, Hefei University of Technology, Hefei, 230009 Anhui China; 4 Animal and Bioscience Research Department, Animal & Grassland Research and Innovation Centre, Teagasc, Grange, Co. Meath, Ireland; 5 Shenzhen Environmental Science and New Energy Technology Engineering Laboratory, Tsinghua-Berkeley Shenzhen Institute, Shenzhen, 518055 People’s Republic of China; 6 Department of Science, Waterford Institute of Technology, Waterford, Ireland

**Keywords:** Co-digestion, Correlation analysis, Dry digestion, Food waste, Hydrogenotrophic methanogenesis, Inoculum, Pig manure, Substrate, Syntrophic oxidation

## Abstract

**Background:**

With the increasing global population and increasing demand for food, the generation of food waste and animal manure increases. Anaerobic digestion is one of the best available technologies for food waste and pig manure management by producing methane-rich biogas. Dry co-digestion of food waste and pig manure can significantly reduce the reactor volume, capital cost, heating energy consumption and the cost of digestate liquid management. It is advantageous over mono-digestion of food waste or pig manure due to the balanced carbon/nitrogen ratio, high pH buffering capacity, and provision of trace elements. However, few studies have been carried out to study the roles of and interactions among microbes in dry anaerobic co-digestion systems. Therefore, this study aimed to assess the effects of different inocula (finished digestate and anaerobic sludge taken from wastewater treatment plants) and substrate compositions (food waste to pig manure ratios of 50:50 and 75:25 in terms of volatile solids) on the microbial community structure in food waste and pig manure dry co-digestion systems, and to examine the possible roles of the previously poorly described bacteria and the interactions among dry co-digestion-associated microbes.

**Results:**

The dry co-digestion experiment lasted for 120 days. The microbial profile during different anaerobic digestion stages was explored using high-throughput 16S rRNA gene amplicon sequencing. It was found that the inoculum factor was more significant in determining the microbial community structure than the substrate composition factor. Significant correlation was observed between the relative abundance of specific microbial taxa and digesters’ physicochemical parameters. Hydrogenotrophic methanogens dominated in dry co-digestion systems.

**Conclusions:**

The possible roles of specific microbial taxa were explored by correlation analysis, which were consistent with the literature. Based on this, the anaerobic digestion-associated roles of 11 bacteria, which were previously poorly understood, were estimated here for the first time. The inoculum played a more important role in determining the microbial community structure than substrate composition in dry co-digestion systems. Hydrogenotrophic methanogenesis was a significant methane production pathway in dry co-digestion systems.

**Electronic supplementary material:**

The online version of this article (10.1186/s13068-018-1344-0) contains supplementary material, which is available to authorized users.

## Background

According to the Irish EPA report, about 390, 279 and 74 kton of biodegradable municipal waste (BMW), primarily comprising food waste (FW), was disposed of by landfilling, composting and anaerobic digestion, respectively, in 2016 in Ireland. The amount treated by anaerobic digestion accounted for only 10%. The EU Landfill Directive (1999/31/EC) requires a diversion of BMW from landfill sites, and the Irish government increased the landfill levy from €30/ton of waste disposed in 2010 to €50/ton in 2011, €65/ton in 2012 and further to €75/ton in 2013 [1]. This provides a good opportunity for anaerobic digestion to be adopted for FW management by the industry.

Annually, about 3.19 million m^3^ of liquid pig manure (PM) is generated in Ireland [2]. Currently, land application is the widely used method for PM management and it is welcomed by silage farmers due to its high nitrogen and phosphorus contents. While, according to the EU Nitrates Directive (91/676/EEC), land application of manure must not be over 170 kg organic nitrogen per hectare per year. It is, therefore, becoming difficult for pig farmers to find suitable lands nearby for disposing of their PM. Hence, it is urgent to find alternative approaches to manage PM in a sustainable and economic way.

Besides landfill and nitrate directives, energy recovery from renewable sources is another important target for the member states to meet in EU. In 2016, the contribution of renewable energy to gross final consumption (GFC) was 9.5% in Ireland, just over halfway towards the 2020 target of 16% in the Directive 2009/28/EC [3]. The contributions of renewable sources to electricity, transport and heating were 27.2%, 6.8% and 5.0%, respectively, still a long way from the 2020 targets of 40%, 10% and 12% [3]. The high organic matter contents of FW and PM make them suitable for anaerobic digestion with the purposes of methane-rich biogas production and waste management. O’Shea et al. [4] studied the biomethane potential of waste substrates in Ireland, including animal manure, household organic waste, milk processing waste and slaughterhouse waste, and estimated that the total biomethane resource could replace the usage of 7.6% natural gas, 7% transport energy, 26.5% industrial gas, or 52% residential gas. Therefore, anaerobic digestion of FW and PM can greatly contribute to meeting EU and Irish targets for increasing renewable energy production, mitigating greenhouse gas (GHG) emissions, diverting MSW from landfilling, and meeting the Nitrates Directive [5, 6].

Neither FW nor PM is suitable for mono-digestion. Zhang et al. [7] reported a low specific methane yield (SMY) of 187 mL/g VS_added_ from mono-digestion of PM due to ammonia inhibition, and a failure of methane production from mono-digestion of FW because of volatile fatty acids (VFA) inhibition; but when PM was co-digested with FW at the ratio of 17:83, the SMY increased to 388 mL/g VS_added_. Kaparaju and Rintala [8] also reported when PM and potato waste were co-digested at the ratio of 80:20, the SMY of 0.30–0.33 m^3^/kg VS_added_ was much higher than that of 0.13–0.15 m^3^/kg VS_added_ obtained in mono-digestion of PM. Wet co-digestion of FW/PM has synergistic effects due to the buffering effect of ammonia and VFA, optimization of the carbon–nitrogen ratio (C/N) and the presence of trace metals in PM [9, 10]. Compared with wet co-digestion at the total solids (TS) content of 3%, the digester volume of dry co-digestion at the TS content of 20% can be decreased by 85%. Therefore, dry digestion can significantly reduce the capital cost and energy consumption in heating, and reduce the cost of digestate liquid management [11].

Inoculum and substrate are important factors affecting the performance of anaerobic digesters. The selection of an appropriate inoculum and selection of appropriate substrate composition can greatly reduce start-up time, improve digestion efficiency and optimize the microbial community structure [12]. Generally, two types of biomass can be added into FW/PM dry digesters as inoculum: one is the dewatered anaerobic sludge obtained in wastewater treatment plants and the other is the finished digestate taken from FW/PM digesters. Using finished digestate taken from FW/PM dry co-digestion digesters as inoculum may improve system efficiency and stability, as its microbiome should be optimized for this environment. Different FW/PM ratios also play a role in microbiome selection because FW/PM ratio determines the C/N ratios, trace element concentrations, VFA and ammonia buffering capacity, etc. However, how inoculum or substrate ratio affects the microbial community structure in dry FW/PM digesters has not yet been studied.

Four stages are included in anaerobic digestion: hydrolysis, acidogenesis, acetogenesis and methanogenesis, and each stage has distinct microbes associated [13]. However, to date, most studies on microbiota of anaerobic digesters have focused on the microbial community structure in stable systems [14, 15], while changes to and development of the microbiome and their possible functionality during different stages in dry co-digestion systems have not been clearly described. In comparison with wet co-digestion systems, dry co-digestion systems would be exposed to extremely high VFA (up to 48.8 g/L) and ammonium (up to 7.3 g/L) concentrations [2]. The physiological characteristics and ecological functions of previously poorly understood dry co-digestion-associated microbes in such harsh conditions are of great interest.

Therefore, the primary objectives of this study were: (1) to investigate the effects of inoculum type (digestate and dewatered anaerobic sludge) and substrate ratio [FW/PM ratios of 50:50 and 75:25 based on volatile solids (VS)] on the microbial community structure during dry co-digestion of FW and PM, and (2) to explore the potential roles of microbes whose functions in dry co-digestion systems are previously poorly described. The 16S rRNA gene amplicon sequencing was employed to investigate the effects of these two factors on the development of microbial community structure within FW/PM dry co-digestion systems. The dry co-digestion experiment lasted for 120 days to determine the microbial profile during different anaerobic digestion stages.

## Methods

### Experimental design and parameter analysis

Batch dry co-digestion of FW and PM was conducted in 1-L glass digesters. Pig manure was collected from a local pig farm in Co. Galway, Ireland. Before use, the PM was centrifuged at 1500×*g* for 5 min (MSE super minor centrifuge, London, UK) and the solid fraction was used. Food waste was collected from 10 local residents and ground to less than 2 mm by a food processor (Kenwood FPP210 Multipro Food Processor, Havant, UK) prior to use. Two inocula were selected: (1) digestate obtained from finished laboratory-scale dry digesters digesting FW/PM, and (2) dewatered anaerobic sludge collected from a Galway wastewater treatment plant, Ireland. The characteristics of FW, PM, digestate and sludge are shown in Table 1.

**Table 1:** Characteristics of substrates and inocula

Characteristics	Digestate	Sludge	Food waste	Pig manure
pH	8.94	8.18	4.98	8.60
Total solids (TS, %)	16.6	18.9	26.2	23.7
Volatile solids (VS, %)	11.6	12.5	25.0	19.4
Soluble chemical oxygen demand (SCOD, g/L)	150.0	38.5	28.4	14.5
Volatile fatty acid (VFA, mg/L)	0	1758	4657	5314

A previous study showed that when using anaerobic sludge as the inoculum, the FW/PM ratio of 50:50 was the preferable operation condition for dry co-digestion of FW and PM (data not shown). Even though a higher SMY was obtained at the FW/PM ratio of 75:25, the lag phase was doubled. Using digestate from existing FW/PM dry co-digestion systems as inoculum was expected to be more resistant to high VFA concentrations, leading to a more stable digestion system and higher SMY. Therefore, the FW/PM ratios of 50:50 and 75:25 were selected based on VS, and totally four conditions were evaluated, denoted R1 (digestate as inoculum, FW/PM = 50:50), R2 (digestate as inoculum, FW/PM = 75:25), R3 (sludge as inoculum, FW/PM = 50:50) and R4 (sludge as inoculum, FW/PM = 75:25). Each condition was replicated four times; consequently, a total of 16 digesters were used. The experimental design is detailed in Table 2. After feeding of the substrates and inocula, tap water was added to digesters to adjust the TS content to 20%. All of the digesters were incubated at 37 °C and shaken by hand once daily. The digesters were operated for 120 days until no more biogas was produced.

**Table 2:** Experimental design of dry co-digestion of food waste and pig manure

Condition	Reactor	Inoculum	FW/PM (VS basis)	Digestate (g)	Sludge (g)	FW (g)	PM (g)	Sampling
R1	1, 2	Digestate	50:50	522.2	–	122.0	157.3	Yes
	3, 4							No
R2	5, 6	Digestate	75:25	534.6	–	186.8	80.8	Yes
	7, 8							No
R3	9, 10	Sludge	50:50	–	508.2	128.4	165.6	Yes
	11, 12							No
R4	13, 14	Sludge	75:25	–	520.2	197.5	84.2	Yes
	15, 16							No

Biogas was collected from all the four replicate digesters under each condition, and samples (~ 1 g) were collected weekly from two replicates of each condition for analysis of TS, VS, soluble chemical oxygen demand (SCOD), total VFA and total ammonia nitrogen (TAN). The two un-sampled replicates of each condition were used to assess the effect of decreasing substrate mass (due to digestate sampling) on methane production. The reduction of VS mass caused by sampling was subtracted while calculating the SMY. Biogas was collected using Tedlar bags; the volume was measured by a flow meter (FMA-1620A-TOT, Omega, Deckenpfronn, Germany) and converted into standard temperature and pressure. Gas chromatography (GC 7890 A, Agilent Technology, Santa Clara, CA, USA) was used to measure the methane content using helium gas as the carrier gas. The TS and VS contents were measured using standard method [16]. The sample was diluted tenfolds by adding 9 parts of deionized water (w/w) and mixing well, and then the pH was measured using a pH meter (pH 3210, WTW, Weilheim, Germany). The dilution was centrifuged at 18,000×*g* for 10 min; the supernatant was filtered through 0.45 μm filter paper before the filtrate was measured for SCOD, total VFA and TAN. The SCOD was measured using standard method [16]. High-performance liquid chromatography (HPLC, Agilent 1200, Agilent Technology, Richardson, TX, USA) was used to analyze total VFA. The standard sample was an equimolar (10 mmol/L) mixture of acetic, propionic, butyric, isobutyric, valeric and isovaleric acids (Sigma-Aldrich, St. Louis, MO, USA). While calculating the total VFA concentration, all the other acids were converted to acetic acid equivalents. The TAN was measured using a nutrient analyzer (Thermo Clinical Labsystems, Vantaa, Finland). COD_VFA_ is the COD equivalent of VFA, which is 1.07 g COD/g acetic acid. COD_VFA + CH4_ is the sum of the COD equivalents of VFA and methane. The COD equivalent of methane is 4 g COD/g CH_4_.

### Analysis of microbe populations by 16S rRNA gene sequencing

Digestate samples (~ 2 g) were taken from the digesters on days 2, 17, 31, 50, 71, 93 and 120 from two replicate digesters under each condition (56 samples in total), snap-frozen in liquid nitrogen and stored at − 80 °C for subsequent microbiota analysis using 16S rRNA gene amplicon sequencing. Frozen digestate (1–2 g) was crushed to a fine powder using a pestle and mortar under liquid nitrogen. Three hundred mg of this frozen powder was then weighed into a frozen (liquid nitrogen) 2 mL cryotube containing Zirconia beads (0.3 g of 0.1 mm and 0.1 g of 0.5 mm, Biospec Products Inc. Bartlesville, OK, USA). Heated extraction buffer (70 °C) was then added to the powder and DNA was extracted using a repeat bead beating method [17].

Modified 16S rRNA gene Illumina adapter fusion primers were used to generate amplicon libraries. The primers were CaporasoNexF 5′TCGTCGGCAGCGTCAGATGTGTATAAGAGACAG[GTGCCAGCMGCCGCGGTAA]3′ and CaporasoNexR 5′GTCTCGTGGGCTCGGAGATGTGTATAAGAGACAG[GGACTACHVGGGTWTCTAAT]3′. The primer sequences outside the square brackets are partial Illumina adapters. The primer sequences inside the square brackets bind to the hypervariable (V4) region of the 16S rRNA gene in bacteria and archaea and are derived from the 16S binding sites of primers previously described by Caporaso et al. [18]. PCR was conducted using 20 ng of digestate DNA as a template and Kapa HiFi Hotstart ReadyMix (Kapa Biosystems, London, UK) according to the manufacturer’s instructions. PCR conditions were: one cycle of 95 °C for 3 min, then 26 cycles of 95 °C for 30 s, 55 °C for 30 s, 72 °C for 30 s, followed by one cycle of 72 °C for 5 min. Amplicons were purified using the QIAquick PCR Purification Kit (Qiagen, Manchester, UK), eluted in 30 µL of buffer EB, and then measured for purity and quantity on a Nanodrop 1000. Two unique 8 bp indices were then added (one index at the 5′ end of the amplicon and the other at the 3′ end) to each amplicon in a second round of PCR using primers from the Illumina Nextera XT indexing kit. PCR was performed with 5 µL of each amplicon as a template and Kapa HiFi Hotstart ReadyMix. PCR conditions for this second round of PCR were: one cycle of 95 °C for 3 min, then 8 cycles of 95 °C for 30 s, 55 °C for 30 s, 72 °C for 30 s, followed by one cycle of 72 °C for 5 min. Indexed libraries were then purified using the Qiagen MinElute PCR Purification Kit (Qiagen, Manchester, UK), eluted in 18 µL of buffer EB, quantified on a Nanodrop1000, then combined in equal concentrations into 2 pools. Each pool was agarose gel-purified to remove primer/adapter dimers using the QIAquick Gel Extraction Kit (Qiagen, Manchester, UK), with an extra purification step used to remove residual agarose. The two pools of gel-purified libraries were then measured for purity and quantity on the Nanodrop 1000 and further quantified using the KAPA SYBR FAST Universal qPCR kit with Illumina Primer Premix (Kapa Biosystems, London, UK). The library pools were then diluted to 2 nM and denatured according to the Illumina MiSeq library preparation guide. 6 pM amplicon library was spiked with 30% denatured and diluted PhiX Illumina control library version 3 (12.5 pM). Two sequencing runs (one library pool per run) were conducted on the Illumina MiSeq using 500 cycle (2 × 250 bp) MiSeq reagent kits (version 2) (Illumina, San Diego, CA, USA).

Reads from all samples were assessed to identify and remove sequencing adaptors and contiguous low-quality bases using the bbmap package (BBMap—Bushnell B.—sourceforge.net/projects/bbmap/). Overlapping reads for each sample were merged using bbmerge (BBMap—Bushnell B.—sourceforge.net/projects/bbmap/) and amplicons of 292 bp (± 1 SD) were retained. The open reference calling method, implemented within the Quantitative Insights Into Microbial Ecology (QIIME) software package, was used to generate operational taxonomic units (OTUs) across all samples. Sequences were clustered at a default similarity level of 97% and a single representative sequence from each OTU was used to align to the Greengenes database (version: gg_13_8) [19]. Taxonomic classification for each OTU was determined with the Ribosomal Database Project (RDP) Classifier using a minimum confidence cut-off of 0.8. OTUs with < 100 sequences summed across all samples were removed from the analysis.

### Statistical analysis

Alpha diversity metrics, for both microbial richness and diversity, were calculated in QIIME1 for the Chao1 no-parametric richness estimator [20], Shannon diversity index [21], Observed Species, and PD Whole Tree [22] using a minimum sample read depth of 51,000 reads. Statistical analysis was performed using R (version 3.3.2) and SPSS 22.0 (IBM, USA). The Shapiro–Wilk test was used to analyze the normality of microbial richness, diversity and phylum level microbial relative abundance, with *P* > 0.05 indicating normal distribution. Microbial richness, diversity and phylum level relative abundance across three different phases [Phases I to III, determined based on the daily specific methane yield (DSMY) and COD_VFA_ as outlined below] and the four operating conditions (R1 to R4) were compared using the Kruskal–Wallis test, with the following pairwise comparison being conducted by the Dunn–Bonferroni post hoc test. Bonferroni correction was used to control the family-wise error rate (FWE) and the adjusted *P* values were used in the results. Comparisons between the two inocula and the two FW/PM ratios were performed by Mann–Whitney U Test. Correlations between genus-level relative abundance and digesters’ physicochemical parameters were performed using a two-tailed Spearman’s rank order correlation. Significant differences and correlations were indicated by *P* < 0.05. QIIME was used to generate the principal coordinate analysis (PCoA) figures for both the weighted and unweighted uniFrac distances, and PERMANOVA was conducted to assess the difference across different conditions.

## Results and discussion

### Operational performance of the digesters

**Fig. 1:**
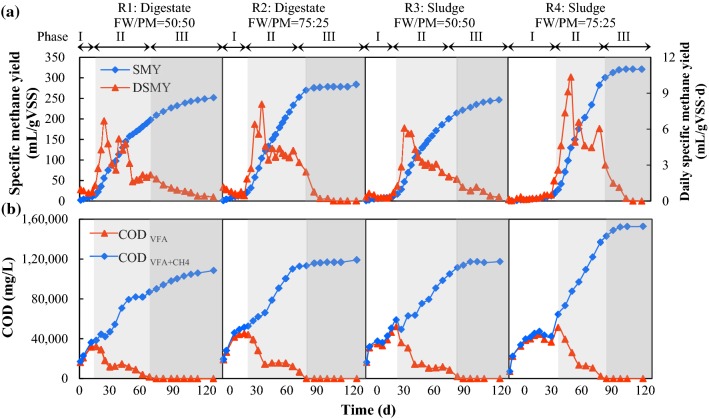
Performance of food waste/pig manure dry co-digestion systems. **a** Specific methane yield (SMY) and daily specific methane yield (DSMY) and **b** COD_VFA_ and COD_VFA+CH4_. SMY and DSMY values are the mean of data from four replicate digesters except at the FW/PM ratio of 75:25, as one of the four replicate digestate inoculum systems and three of the four replicate sludge inoculum systems were inhibited, with almost no methane production. Only the results from the uninhibited digesters are shown here. COD_VFA_ and COD_VFA + CH4_ values are the mean of data from duplicate digesters where samples were taken

The profiles of methane production, COD_VFA_ concentration and COD_VFA+CH4_ concentration are shown in Fig. 1. According to the DSMY data and COD_VFA_ concentration, the dry co-digestion process could be divided into three phases. Phase I was the lag phase, during which COD_VFA_ increased rapidly and there was almost no methane production. In Phase II, COD_VFA_ decreased and almost 80% of the methane yield was produced during this period. Hydrolysis and acidification continued as COD_VFA + CH4_ continued to increase. In phase III, all of the COD_VFA_ was consumed and a reduced volume of methane was produced.

As described previously [2], at a FW/PM ratio of 50:50, there was no significant difference between the SMY in the digestate (252 mL/gVS_added_) and sludge (246 mL/gVS_added_) inoculum systems (*P* > 0.05). However, using digestate as inoculum resulted in a considerable decrease in the lag phase (13 days) compared with the sludge inoculum systems (28 days). At the FW/PM ratio of 75:25, the methane production ceased on Day 40 in one of the four replicate digestate inoculum systems, with a total SMY of only 22 mL/g VS_added_ at the end of the experiment; and no methane was produced since day 33 in three of the four replicate sludge inoculum systems, with the total SMYs of only 6–7 mL/g VS_added_ at the end of the experiment. It indicated that these digesters were severely inhibited. A similar trend was observed by Abbassi-Guendouz et al. [23]: at the TS content of 30%, two replicates in four had similar methane production to those at 25% TS and the other two were inhibited as those at 35% TS. Mass transfer limitation at high TS content was considered to cause it. A previous study indicated high VFA concentrations were the main inhibition factors for methane production during dry co-digestion of FW and PM [2]. A high FW/PM ratio resulted in rapid accumulation of VFAs, which may reach the critical tolerance of the microbes. If the VFA-consuming bacteria and methanogens in the digesters were sufficient and resistant enough to stress conditions, VFAs might be utilized in time and methane could be produced properly. Otherwise, inhibition happened. Using digestate as inoculum improved the stability of the dry co-digestion systems. It may be because the digestate inoculum had been acclimated in FW/PM dry co-digestion systems and developed predominant microorganisms, while the sludge inoculum had to undergo an adaptation and selection period, which decreased its competitiveness. As a result, digestate as inoculum and a FW/PM ratio of 50:50 were recommended as preferable operation conditions.

### Microbial richness and diversity

**Fig. 2:**
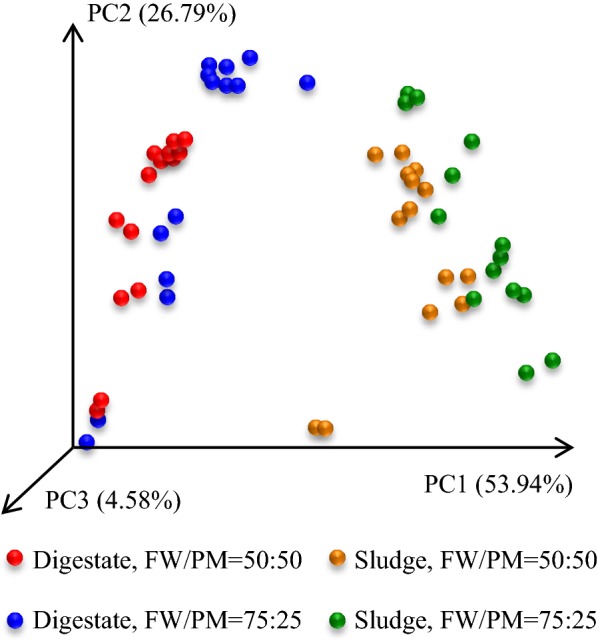
Principal coordinate analysis (PCoA; based on weighted UniFrac distances) of microbial community structure in dry digesters co-digesting food waste and pig manure

The number of reads per sample ranged from 57,866 to 222,595 across the 56 samples taken from the digesters. A total of 1987 OTUs were found (1934 *Bacteria* and 53 *Archaea*), with 147 of them (138 *Bacteria* and 9 *Archaea*) representing 80% of the total reads. Similarly, Kirkegaard et al. [24] studied the microbial community composition in 32 full-scale anaerobic digesters, and found 300 OTUs represented 80% of the total reads across all plants. PCoA was performed to analyze beta diversity across the digestate samples, and clear distinction was observed between the sludge and digestate inoculum systems in Fig. 2. A straight PERMANOVA across all conditions was significant at *P* < 0.001 level, indicating a significant difference between the groups. The detailed differences in microbial richness and diversity caused by inoculum and FW/PM ratio were further described below.

Alpha diversity metrics were used to assess microbial richness (Observed Species and Chao1 Index) and diversity (Shannon Index and PD Whole Tree) within the digestate samples, with the results and statistical analysis shown in Table 3. The Chao1 Index, Observed Species and PD Whole Tree in Phase II and III were significantly higher than those in Phase I (*P* < 0.01), but did not differ between Phase II and Phase III (*P* > 0.05). It implies that both the microbial richness and diversity increased with time during the dry co-digestion process. Significant differences in the microbial diversity and richness were observed between different operating conditions (*P* < 0.01). Both inoculum and FW/PM ratio contributed to the differences, but the diversity differences (PD Whole Tree and Shannon Index) were mainly influenced by the inoculum (*P* < 0.01), while the richness differences (Chao1 Index and Observed Species) were mainly influenced by the FW/PM ratio (*P* < 0.01). At the same FW/PM ratio, significant differences were observed between different inocula: *P* < 0.05 for PD Whole Tree and *P* < 0.001 for Shannon Index between R1 and R3 (FW/PM = 50:50), and *P* < 0.01 for Shannon Index between R2 and R4 (FW/PM = 75:25). At the same inoculum, the only significant difference was observed in Observed Species (*P* < 0.05) between R3 and R4 in sludge inoculum systems. It indicated that the inoculum played a more important role in determining the microbial community structure than substrate composition in dry co-digestion systems. Apart from being introduced from inocula, dominant microbes can also be accumulated from substrates alone. For instance, Abendroth et al. [25] reported the accumulation of *Firmicutes* and *Bacteroidetes* from a separate hydrolysis of grass, and Barret et al. [26] reported the accumulation of *Methanoculleus* from anoxic storage of swine manure. However, accumulation of dominant microbes directly from substrates may cause VFA inhibition and it may take a long time to accumulate methanogens. As mentioned above, the proper selection of inoculum can introduce acclimated competitive microbes directly, which can greatly improve the system stability and reduce the lag phase.

**Table 3:** Microbial richness and diversity at the genus level during dry co-digestion of food waste and pig manure [values are the mean of data from duplicate reactors ± standard deviation (SD)]

	Richness		Diversity	
	Chao1	Observed species	PD whole TREE	Shannon
Phase I	1415 ± 125	1208 ± 126	92.8 ± 8.4	6.58 ± 0.93
Phase II	1595 ± 72	1366 ± 64	102.2 ± 3.9	6.85 ± 0.69
Phase III	1637 ± 54	1404 ± 83	104.8 ± 4.6	6.97 ± 0.58
R1	1597 ± 123	1312 ± 112	98.7 ± 7.2	6.09 ± 0.41
R2	1570 ± 118	1294 ± 123	97.5 ± 7.6	6.30 ± 0.49
R3	1582 ± 122	1411 ± 124	104.9 ± 7.6	7.47 ± 0.41
R4	1473 ± 111	1310 ± 93	99.8 ± 5.5	7.37 ± 0.31

	Statistical analysis (*P* values)			
	Chao1	Observed species	PD whole tree	Shannon
Phase	0.000***	0.000***	0.000***	0.667
Phase I vs Phase II	0.001***	0.002**	0.008**	1.000
Phase I vs Phase III	0.000***	0.000***	0.000***	1.000
Phase II vs Phase III	0.500	0.927	0.424	1.000
Condition	0.008**	0.004**	0.002**	0.000***
Inoculum	0.057	0.022*	0.008**	0.000***
FW/PM	0.007**	0.009**	0.013*	0.941
R1 vs. R2	1.000	1.000	1.000	1.000
R1 vs. R3	1.000	0.078	0.048*	0.000***
R1 vs. R4	0.007**	1.000	1.000	0.000***
R2 vs. R3	1.000	0.003**	0.002**	0.000***
R2 vs. R4	0.309	1.000	1.000	0.007**
R3 vs. R4	0.069	0.039*	0.070	1.000

*** *P* < 0.001, ** *P* < 0.01, * *P* < 0.05

### Microbial community composition at the phylum level

The phylum-level relative abundances for microbial communities at four different operating conditions are shown in Additional file 1: Fig. S1. Nine abundant phyla (relative abundance > 5% in at least one sample) were found in all reactors. In the digestate inoculum systems, *Firmicutes* (41.9–56.1% relative abundance), *Bacteroidetes* (10.9–46.7%) and *Euryarchaeota* (0.9–11.5%) were the most abundant phyla. While in the sludge inoculum systems, the most abundant phyla were *Firmicutes* (26.2–45.2%), *Proteobacteria* (10.6–22.9%), *Bacteroidetes* (4.4–19.8%) and *Euryarchaeota* (2.3–16.0%).

Differences were observed between the different operating conditions for both bacterial (*P* < 0.01) and *Archaeal* (*P* < 0.05) phyla (Table 4). However, the differences for bacteria were mainly caused by the different inocula (*P* < 0.01), while the differences in archaea resulted from the different FW/PM ratios (*P* < 0.05). There were no significant differences within the same inoculum type (R1 vs. R2 and R3 vs. R4; *P* > 0.05), indicating the lack of effect of the FW/PM ratio. The relative abundances of *Firmicutes*, *Bacteroidetes* and *Thermotogae* were significantly higher in the digestate inoculum systems (*P* < 0.01), while relative abundances of *Actinobacteria*, *Chloroflexi*, *Proteobacteria*, *Synergistetes* and *WWE1* were significantly higher in the sludge inoculum systems (*P* < 0.001). Changes to abundances of *Euryarchaeota* were mainly in response to the reaction phase (*P* < 0.001); the abundance increased over time, being significantly higher in Phase III than in Phase II (*P* < 0.05) or Phase I (*P* < 0.001).

**Table 4:** Differences in microbial composition at the phylum level during dry co-digestion of food waste and pig manure using four different operating conditions (mean ± SD)

Relative abundance (%)	R1 (digestate, FW/PM = 50:50)	R2 (digestate, FW/PM = 75:25)	R3 (sludge, FW/PM = 50:50)	R4 (sludge, FW/PM = 75:25)
*Euryarchaeota*	5.0 ± 2.2	7.9 ± 4.0	4.0 ± 1.8	7.4 ± 5.2
*Actinobacteria*	2.5 ± 0.3	2.8 ± 0.5	6.3 ± 1.2	5.9 ± 1.3
*Bacteroidetes*	28.9 ± 8.8	18.5 ± 10.5	10.9 ± 5.0	7.6 ± 2.0
*Chloroflexi*	1.1 ± 0.3	1.0 ± 0.4	7.5 ± 2.3	7.5 ± 2.8
*Firmicutes*	49.9 ± 4.7	52.4 ± 2.5	39.4 ± 7.4	33.7 ± 8.0
*Proteobacteria*	4.4 ± 0.5	6.0 ± 1.1	14.8 ± 2.3	18.3 ± 5.3
*Synergistetes*	2.4 ± 0.3	3.1 ± 0.4	7.0 ± 1.5	6.7 ± 1.1
*Thermotogae*	4.2 ± 1.5	5.9 ± 3.3	2.6 ± 1.3	3.5 ± 1.0
*WWE1*	0.3 ± 0.1	0.3 ± 0.2	2.2 ± 1.1	3.4 ± 1.2

Groups	Statistical analysis (*P* values)												
	Condition	Inoculum	FW/PM	R1 vs R2	R1 vs R3	R1 vs R4	R2 vs R3	R2 vs R4	R3 vs R4	Phase	Phase I vs II	Phase I vs III	Phase II vs III
*Euryarchaeota*	0.040*	0.149	0.014*	0.945	1.000	1.000	0.035*	1.000	0.235	0.000***	0.003**	0.000***	0.016*
*Actinobacteria*	0.000***	0.000***	0.706	1.000	0.000***	0.000***	0.000***	0.001***	1.000	0.725	1.000	1.000	1.000
*Bacteroidetes*	0.000***	0.000***	0.019*	0.235	0.000***	0.000***	0.228	0.005**	1.000	0.023*	0.669	0.019**	0.229
*Chloroflexi*	0.000***	0.000***	0.935	1.000	0.000***	0.000***	0.000***	0.000***	1.000	0.157	1.000	1.000	1.000
*Firmicutes*	0.000***	0.000***	0.922	1.000	0.007**	0.000***	0.000***	0.000***	1.000	0.030*	0.264	0.025*	0.715
*Proteobacteria*	0.000***	0.000***	0.039*	0.278	0.000***	0.000***	0.012*	0.000***	1.000	0.276	0.320	0.105	0.490
*Synergistetes*	0.000***	0.000***	0.195	0.171	0.000***	0.000***	0.002**	0.007**	1.000	0.983	1.000	1.000	1.000
*Thermotogae*	0.003**	0.001**	0.105	1.000	0.099	1.000	0.003**	0.119	1.000	0.002**	0.004**	0.006**	1.000
*WWE1*	0.000***	0.000***	0.251	1.000	0.001***	0.000***	0.001***	0.000***	0.866	0.265	1.000	1.000	1.000

Only phyla with a relative abundance > 5% in at least one sample are shown

*** *P* < 0.001, ** *P* < 0.01, * *P* < 0.05

### Correlations between bacterial taxa and digesters’ physicochemical parameters

Bacteria play significant roles in hydrolysis, acidogenesis and acetogenesis in anaerobic digestion systems. However, the possible roles of many of the resident bacteria have not been elucidated. In this study, correlation analysis between the relative abundance of the dominant bacterial taxa and digesters’ physicochemical parameters over the 120-day operating period was performed to explore the possible microbial roles (Table 5). The physicochemical parameters included: SCOD, total VFA, free VFA, acetate, propionate, butyrate and SMY. The main findings of the correlation analysis are summarized in the sections below.

**Table 5:**
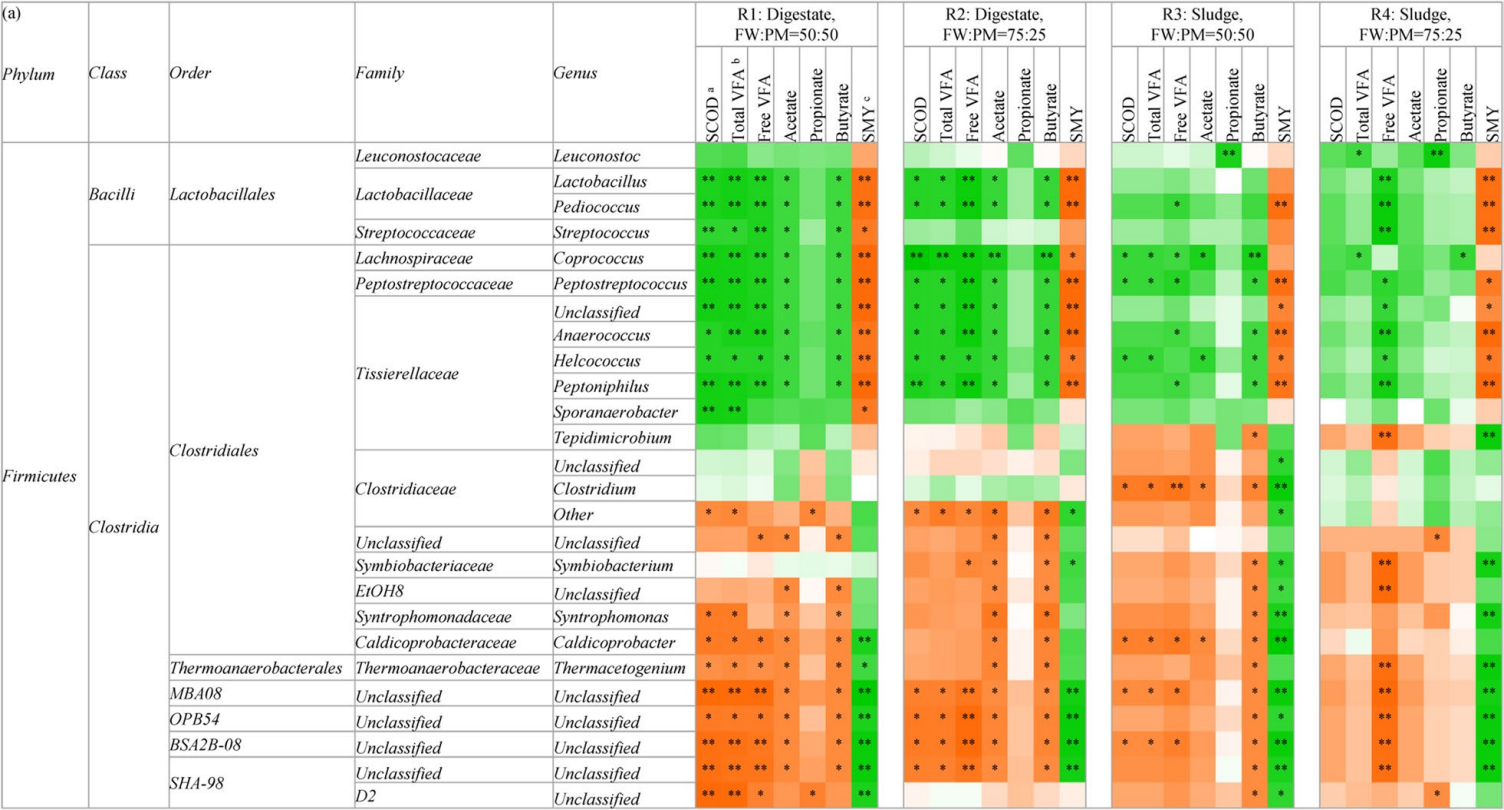

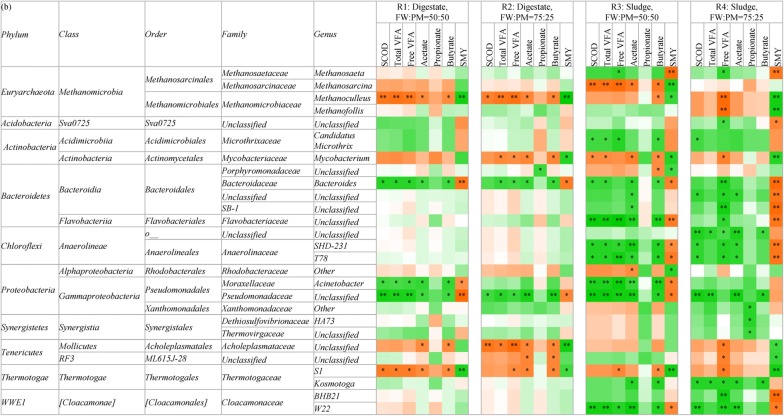
Correlations between the relative abundance of microbial taxa and physicochemical parameters during dry co-digestion of food waste and pig manure under the four different operating conditions: (a) *Firmicutes* and (b) other phyla

The relative abundance and physicochemical parameters are the mean of data from the duplicate digesters sampled from R1 to R3, and are the data from the uninhibited digester from R4

Red boxes indicate negative correlations, green boxes indicate positive correlations, and blank boxes indicate no correlations

Correlations were determined using a two-tailed pairwise Spearman’s rank order correlation at a significance level of *P* < 0.05

* *P* < 0.05; ** *P* < 0.01

^a^SCOD: Soluble chemical oxygen demand

^b^VFA: Volatile fatty acid

^c^SMY: Specific methane yield

*Firmicutes* are prevalent in co-digestion systems treating substrates such as restaurant, household and slaughterhouse wastes [27]. Several members are well known as fermentative and syntrophic bacteria [14]. In this study, correlations for *Firmicutes* members were more evident in the digestate inoculum systems than in the sludge inoculum systems (Table 5a). In these systems, the genera *Lactobacillus*, *Pediococcus* and *Streptococcus* in order *Lactobacillale*, and genera *Coprococcus*, *Peptostreptococcus*, *Anaerococcus*, *Helcococcus* and *Peptoniphilus* in order *Clostridiales* had positive correlations with SCOD, total VFA, free VFA, acetate and butyrate, and had negative correlations with SMY. It indicates that members in these genera may play roles in hydrolysis and acidogenesis, which degraded organic matters to SCOD and further converted SOCD into various VFAs, mostly acetate and butyrate. As it is well known that, even within the same species, the metabolic potential of different strains can be in huge differences, the correlations just indicated members working on hydrolysis and acidogenesis might be dominant in these genera. These are corroborated, at least to some extent, when the metabolic traits of these bacteria were reviewed. *Lactobacillus*, *Pediococcus* and *Streptococcus* are well known as lactic acid producers [28], while *Coprococcus* can ferment carbohydrate with the resultant production of acetate, butyrate and other VFA [29]. *Peptoniphilus* and *Anaerococcus* are derived from the genus *Peptostreptococcus*; members of *Peptoniphilus* are reported to be non-saccharolytic, using peptone as a major energy source, while *Anaerococcus* members are reported saccharolytic. They all produce butyrate as a terminal VFA [30]. Two species within the genus *Helcococcus* (*kunzii* and *sueciensis)* produce acids from lactose and trehalose [31], while *Helcococcus ovis* reportedly produces acids from glucose [32].

On the contrary, the genera *Syntrophomonas*, *Caldicoprobacter*, *Thermacetogenium* and some unclassified genera in the candidate orders *MBA08*, *OPB54*, *BSA2B*-*08* and *SHA*-*98* had negative correlations with SCOD, total VFA, free VFA, acetate and butyrate, and had positive correlations with SMY (Table 5a). The members responsible for syntrophic oxidation might be dominant in these orders, which are the main actors consuming VFA, such as acetate, propionate and butyrate, under high ammonia or VFA conditions [33]. Syntrophic oxidations are endergonic reactions (Δ*G*^0^′ > 0) and thermodynamically unfavorable under standard conditions (*P* = 1 atm, *T* = 298 K). These reactions occur only when the products are consumed by hydrogenotrophic methanogens, resulting in low partial pressure of hydrogen and low concentrations of acetate and formate [33]. The dominance of hydrogenotrophic methanogens in FW/PM dry co-digestion FW/PM systems made this possible, as outlined in the methanogen section. The genus *Syntrophomonas* is well known as butyrate-oxidizing bacterium [34], and the species *Thermacetogenium phaeum* isolated from thermophilic digesters was reported to be a syntrophic acetate-oxidizing bacterium [35]. These traits are in agreement with those observed in this study. The genus *Caldicoprobacter* has been reported to be abundant at high TAN concentrations (5.0–25.0 g/L) [36] and in thermophilic conditions [37]. In this study, the TAN concentrations ranged 3.9–7.2 g/L and incomplete mixing in the dry co-digestion reactors may have caused local thermophilic temperatures (hot spots), enabling the existence of *Caldicoprobacter*. Some species of *Caldicoprobacter* isolated from hot springs or sheep’s faeces, such as *algeriensis*, *oshimai* and *guelmensis* reportedly ferment various sugars with the resultant production of acetate, lactate, ethanol, CO_2_, and H_2_ [38, 39]. However, the negative correlations of *Caldicoprobacter* with acetate and butyrate in this study indicate that some species within this genus may function as syntrophic oxidizers of acetate and butyrate, which has not previously been reported. Deng et al. [40] observed a similarly positive correlation of *Caldicoprobacter* with daily methane production and a negative correlation with butyrate, but did not extrapolate the syntrophic oxidation function. The candidate order *MBA08* is mainly observed in thermophilic conditions [41] and, together with the order *SHA*-*98*, also in anaerobic digesters treating agricultural waste [42]. The order *OPB54* was previously found in thermophilic digesters and at high TAN concentrations (7.0 g/L) [43, 44]. But the candidate order *BSA2B*-*08* has not previously been reported in anaerobic digestion systems. Moreover, the possible roles of these candidate orders (*MBA08*, *OPB54*, *SHA*-*98* and *BSA2B*-*08*) in anaerobic digesters have not previously been reported. Their negative correlations with various VFAs (especially acetate and butyrate) and their positive correlation with SMY mean that some members functioning as syntrophic acetate- and butyrate-oxidizing bacteria may dominant in dry co-digestion systems. The candidate family *D2* within the order *SHA*-*98* had a negative correlation with propionate, indicating that it may contain propionate-oxidizing bacteria.

The phylum *Bacteroidetes* includes species active in the hydrolysis and acidogenesis stages of anaerobic digestion [45]. The genus *Bacteroides* predominated in both digestate (6.7–42.5%) and sludge inoculum systems (0.1–16.3%) (Additional file 2: Fig. S2). It had positive correlations with SCOD, total VFA, free VFA, acetate and butyrate, and negative correlations with SMY (Table 5b). This indicates that members working on hydrolysis and acidification might be dominant in *Bacteroides* in dry co-digestion systems. In line with this, *Bacteroides cellulosolvens* has been reported to ferment cellulose and cellobiose to produce acetic acid, ethanol, CO_2_/H_2_ and a little lactic acid [46] and other species of *Bacteroides* can degrade starch [47].

In the phylum *Proteobacteria*, the *Alphaproteobacteria* and *Gammaproteobacteria* classes were abundant. The members in families *Rhodobacteraceae* and *Pseudomonadaceae* dominated and were much higher in sludge inoculum systems than in the digestate inoculum systems (Additional file 2: Fig. S2). The genus *Acinetobacter* and an unclassified genus from the family *Pseudomonadaceae* had positive correlations with SCOD, total VFA, free VFA, acetate and butyrate (Table 5b), indicating members responsible for hydrolysis and acidification might be dominant in these genera, with acetate and butyrate as main products. The functions of these taxa within anaerobic digestion systems have not been clearly reported previously. *Pseudomonas putida* in family *Pseudomonadaceae* reduced the COD by 44.4% during the anaerobic treatment of distillery spent wash [48], and strains within the genus *Acinetobacter* reduced the COD by 44% when anaerobic treatment of molasses spent wash [49]. However, the products of hydrolysis were not reported. Furthermore, Thangaraj et al. [50] reported that among 31 *Acinetobacter* isolates assayed, 11 could utilize aromatic compounds and produce acidic intermediates, but the detailed products were not clear. All these reports support the positive correlations observed between VFA and the relative abundances of family *Pseudomonadaceae* and the genus *Acinetobacter* in this study, but the possible end products of acetate and butyrate were indicated in this study.

The phylum *Chloroflexi* was mainly detected within the sludge inoculum systems, and was reported to be able to utilize glucose [34]. In sludge inoculum systems, the candidate genus *T78* predominated, followed by *SHD*-*231* (Additional file 2: Fig. S2), they both belong to the family *Anaerolinaceae*. These two candidate genera had positive correlations with SCOD, total VFA, free VFA, acetate and butyrate, which indicated the possible hydrolysis and acidification activities of some members (Table 5b). The family *Anaerolinaceae* was previously reported to ferment carbohydrate to produce acetate and H_2_ [51, 52]. The candidate genus *T78* has the potential to decompose carbohydrates [41] and degrade long chain petroleum hydrocarbons [53]. These traits are all in line with the results observed in the current study. However, the detailed function of candidate genus *SHD*-*231* has not yet been reported. Based on the results of this study, some of its members may work on hydrolysis and acidification of organic matter, producing various VFAs, especially acetate and butyrate.

Two genera from the phylum *Thermotogae* predominated within the digesters; the candidate genus *S1* was mainly found in the digestate inoculum systems, and *Kosmotoga* mainly in the sludge inoculum systems (Additional file 2: Fig. S2). The phylum *Thermotogae* is reported to be dominant in thermophilic anaerobic digestion systems [54], which indicated the occurrence of localized hot spots during the dry co-digestion of FW and PM. Positive correlations were observed between the genus *Kosmotoga* and SCOD, total VFA, free VFA, acetate and butyrate (Table 5b), indicating the probable hydrolysis and acidification activities of some members. *Kosmotoga* species are reported to be capable of fermenting carbohydrates, peptides and pyruvate [55], which agrees with the results observed in this study. The role of the candidate genus *S1* has not previously been reported. In the present study, it was negatively correlated with SCOD, total VFA, free VFA, acetate and butyrate, and positively correlated with SMY (Table 5b), indicating some members working on acetate and butyrate syntrophic oxidation might be dominant in this genus.

The candidate phylum *WWE1* is reported to play a role in hydrolysis of cellulose and/or fermentation of hydrolysis products in anaerobic digesters [56]. In this study, *WWE1* was mainly found in the sludge inoculum systems (Additional file 2: Fig. S2). Positive correlations were observed between the candidate genus *W22* and SCOD, total VFA, free VFA, acetate and butyrate (Table 5b), indicating the possible role in hydrolysis and acidogenesis of some members. The role of the candidate genus *W22* in anaerobic digesters has not yet been clearly reported in literature.

Correlation analysis between the relative abundances of specific bacterial taxa and digesters’ physicochemical parameters provides a qualitative analysis method to explore the possible roles of and the interactions among these microbes. The high consistence of the roles explored in this study with findings reported in the literature indicates this method is effective and instructive to some extent. By this way, the possible roles of 11 bacterial taxa, which were previously poorly described in anaerobic digestion systems, were predicted for the first time in the present study as summarized in Additional file 3: Table S1. These can provide references and possible directions for future investigation of these bacteria in anaerobic digesters. However, predicting the exact function of specific bacterial taxa requires more in-depth studies of microbiology researchers.

**Fig. 3:**
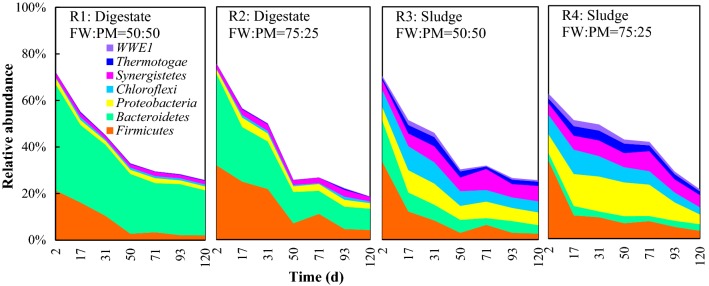
Phylum-level relative abundance of hydrolysis- and fermentation-associated bacteria during dry co-digestion of food waste and pig manure under four different operating conditions. Values are the mean of data from two replicates of each condition at each time point

Almost all of the taxa likely working on syntrophic oxidation belonged to the phylum *Firmicutes*; however, the distribution of hydrolysis- and acidification-associated taxa varied with the different operating conditions (Fig. 3). In the digestate inoculum systems, the phyla *Firmicutes* and *Bacteroidetes* were the main contributors to hydrolysis and acidification; while in the sludge inoculum systems, more phyla contributed, including *Firmicutes*, *Bacteroidetes*, *Proteobacteria*, *Chloroflexi*, *Synergistetes*, *Thermotogae* and *WWE1*.

### Methanogen composition and correlations with digesters’ physicochemical parameters

**Fig. 4:**
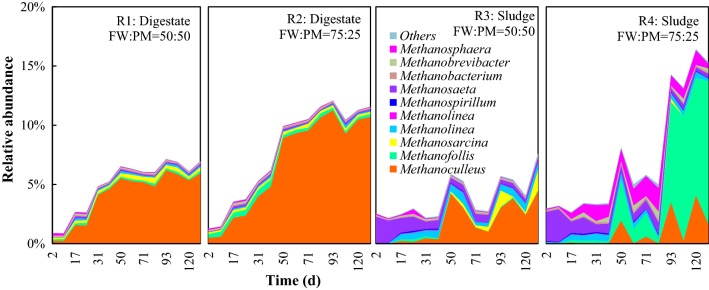
Relative abundance of methanogens at the genus level during dry co-digestion of food waste and pig manure under four different operating conditions. Values are the mean of data from two replicates of each condition at each time point

**Fig. 5:**
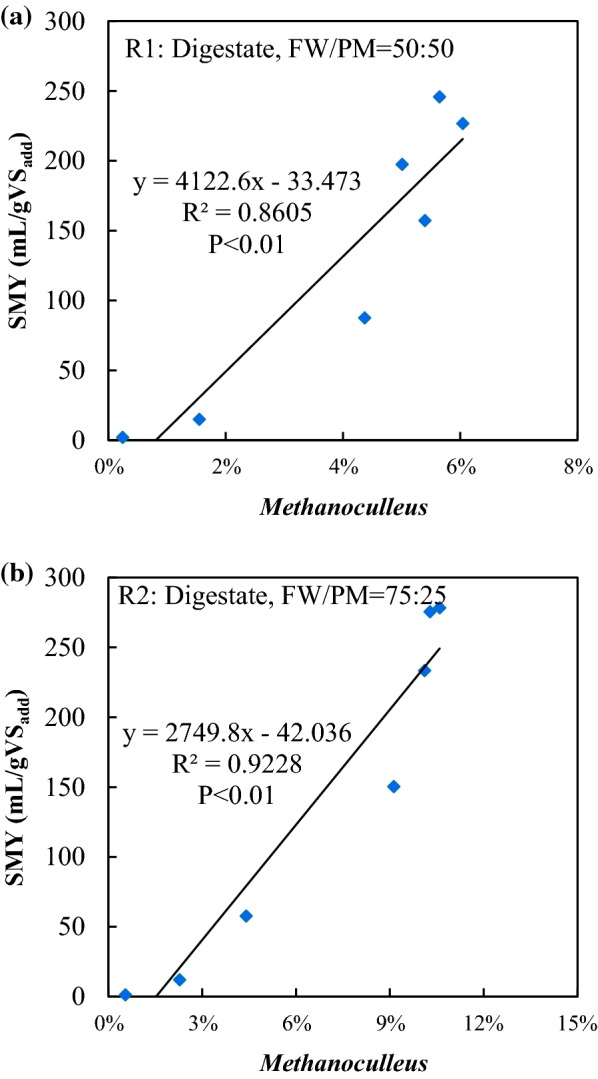
Correlation between the mean relative abundance of *Methanoculleus* and the mean specific methane yield in R1 (**a**) and R2 (**b**)

The relative abundances of methanogens under the different operating conditions are shown in Fig. 4. In the digestate inoculum systems, the genus *Methanoculleus* was dominant, with relative abundances of 5.6% and 10.5% at the end of the experiment at the FW/PM ratios of 50:50 and 75:25, respectively, accounting for 85.3% and 92.6% of the total methanogens. Members of the genus *Methanoculleu*s isolated thus far are hydrogenotrophic methanogens and can utilize H_2_/CO_2_ but not acetate as substrates for methanogenesis [57]. Barret et al. [26] stated that *Methanodulleus* can be used as a biomarker to indicate the methanogenic activity in an anoxic swine manure storage tank, and hydrogenotrophic pathway was the dominant methanogenesis method. It highly agreed with the results obtained in this study. Significant positive correlations were established between the relative abundance of *Methanoculleu*s and SMY in the digestate inoculum systems (Fig. 5, *P* < 0.01). This indicates that *Methanoculleu*s was the main contributor to methane production in these systems.

In the sludge inoculum systems, the methanogen composition was much more diverse and their relative abundance fluctuated more compared with the digestate inoculum systems, indicating the instability of the sludge inoculum systems. The genus *Methanosaeta* dominated at the beginning of the experiment, accounting for 82.3–87.2% of the total methanogens, but almost no methane was produced during this period. The production of methane started to increase when *Methanosaeta* was substituted by *Methanoculleus* and *Methanofolli*s. At the end of the experiment, the proportions of *Methanosaeta* decreased to < 5%. At the FW/PM ratio of 50:50, *Methanoculleus* was abundant in Phase III, accounting for 53.0–70.3% of the total methanogens. At the FW/PM ratio of 75:25, *Methanofollis* and *Methanoculleus* dominated in Phase III, accounting for 59.0–79.9% and 10.8–25.0% of all methanogens, respectively. It indicated that the acetoclastic pathway was inhibited and hydrogenotrophic pathway became the main methane production method.

*Methanosaeta* is an acetoclastic methanogen which uses only acetate as a substrate for methane production [14]. It dominates only at low acetate concentrations and is highly sensitive to changes in environmental conditions [58]. Similar to *Methanoculleus*, *Methanofollis* is a hydrogenotrophic methanogen as well, which can utilize H_2_/CO_2_, formate, methanol, ethanol, 1-propanol, 1-butanol, and trimethylamine but not acetate for growth and methane production [59]. Hydrogenotrophic methanogens are reported more resistant to stress factors compared with acetoclastic methanogens. Calli et al. [60] found that *Methanosaeta* was substituted by *Methanosarcina* as TAN increased from 1.0 to 2.5 g/L. Ziganshin et al. [61] reported that *Methanosaeta* prevailed at low organic loading rates (OLRs) and were outcompeted by *Methanosarcina* at high acetate concentrations and then dominated by *Methanoculleus* with even higher propionate and acetate accumulations. The high VFA (up to 48.8 g/L) and TAN (up to 7.3 g/L) concentrations in FW/PM dry co-digestion systems were selected for more robust hydrogenotrophic methanogens [2]. The substitution of *Methanosaeta* by *Methanoculleus* and *Methanofollis* in the present study agrees with this. The negative correlation between *Methanosaeta* and SMY in the sludge inoculum systems can be explained by the inhibition of *Methanosaeta*, while the positive correlations between *Methanoculleus/Methanofollis* and SMY indicated their major contribution to methane production (Table 5b). Therefore, hydrogenotrophic methanogenesis conducted by *Methanoculleus* and *Methanofollis* was the dominant methane production pathway in FW/PM dry co-digestion systems, with the acetoclastic pathway being inhibited. This result is supported by the observation of syntrophic oxidation bacteria, as discussed in the *Firmicutes* section.

## Conclusions

The effects of inoculum and FW/PM ratio on the microbial community structure during dry co-digestion of FW/PM were studied. The results showed that the inoculum factor was more significant in determining the microbial community structure than the substrate composition factor. Correlation analysis between the relative abundance of specific microbial taxa and physicochemical parameters was performed to provide information on their possible roles and interactions within anaerobic digestion systems. In this way, the dry digestion-associated roles of 11 bacteria whose functions were previously poorly understood were predicted for the first time.

The finding that the inoculum factor played a significant role for a balanced microbial community in the dry digesters indicates that in continuous operations, it would be important to maintain a certain amount of finished digestate in the digesters so as to obtain a healthy microbial community within the digesters. The correlation analysis can provide a proper method to explore the possible roles of microbes in anaerobic digestion systems to some extent before carrying out intensive pure culture analysis. However, the accurate prediction on the function of certain taxa requires more in-depth microbiological studies.

## Additional files

**Additional file 1: Fig. S1.** Phylum-level relative abundance during dry co-digestion of food waste and pig manure using four different operating conditions.

**Additional file 2: Fig. S2.** Genus-level relative abundance of bacteria during dry co-digestion of food waste and pig manure using four different operating conditions.

**Additional file 3: Table S1.** Predicted anaerobic digestion-associated functions of bacterial taxa whose roles have not previously been reported in the literature.

## Abbreviations

FW: food waste
PM: pig manure
GHG: greenhouse gas
SMY: specific methane yields
VFA: volatile fatty acid
C/N: carbon to nitrogen ratio
R1: digestate as inoculum, FW/PM = 50:50
R2: digestate as inoculum, FW/PM = 75:25
R3: sludge as inoculum, FW/PM = 50:50
R4: sludge as inoculum, FW/PM = 75:25
TS: total solids
VS: volatile solids
SCOD: soluble chemical oxygen demand
TAN: total ammonia nitrogen
DSMY: daily specific methane yield
FEW: family-wise error rate
PCoA: principal coordinate analysis
