# Supplementary material for: Exploring the roles of and interactions among microbes in dry co-digestion of food waste and pig manure using high-throughput 16S rRNA gene amplicon sequencing
Source: Biotechnol Biofuels. 2019 Jan 4;12:5. doi: 10.1186/s13068-018-1344-0 (PMC6318937; doi:10.1186/s13068-018-1344-0)
Supplement: Supplementary file 1 — Additional file 1: Fig. S1. Phylum-level relative abundance during dry co-digestion of food waste and pig manure using four different operating conditions. [file 13068_2018_1344_MOESM1_ESM.docx]

# Exploring the roles of and interactions among microbes in dry co-digestion of food waste and pig manure using high-throughput 16S rRNA gene amplicon sequencing

Yan Jiang^1^, Conor Dennehy^1^, Peadar G. Lawlor^2*^, Zhenhu Hu^3^, Matthew McCabe^4^, Paul Cormican^4^, Xinmin Zhan ^1,5*^, Gillian E. Gardiner^6^

^1^ Civil Engineering, College of Engineering & Informatics, National University of Ireland, Galway, Ireland

^2^ Teagasc, Pig Development Department, Animal & Grassland Research and Innovation Centre, Moorepark, Fermoy, Co. Cork, Ireland

^3^ School of Civil Engineering, Hefei University of Technology, Hefei, 230009, Anhui Province, China

^4^ Animal and Bioscience Research Department, Animal & Grassland Research and Innovation Centre, Teagasc, Grange, Co. Meath, Ireland

^5^ Shenzhen Environmental Science and New Energy Technology Engineering Laboratory, Tsinghua-Berkeley Shenzhen Institute, Shenzhen 518055, PR China

^6^ Department of Science, Waterford Institute of Technology, Waterford, Ireland

*Corresponding author

Xinmin Zhan

Phone: +353 91 495239

Notes: The authors declare no competing financial interest.

Fig. S1. Phylum-level relative abundance during dry co-digestion of food waste and pig manure using four different operating conditions. The nine abundant phyla are in bold. Data are from two replicates of each condition at each time point.
