# Supplementary material for: Exploring the roles of and interactions among microbes in dry co-digestion of food waste and pig manure using high-throughput 16S rRNA gene amplicon sequencing
Source: Biotechnol Biofuels. 2019 Jan 4;12:5. doi: 10.1186/s13068-018-1344-0 (PMC6318937; doi:10.1186/s13068-018-1344-0)
Supplement: Supplementary file 2 — Additional file 2: Fig. S2. Genus-level relative abundance of bacteria during dry co-digestion of food waste and pig manure using four different operating conditions. [file 13068_2018_1344_MOESM2_ESM.docx]

Fig. S2. Genus-level relative abundance of bacteria during dry co-digestion of food waste and pig manure using four different operating conditions. Data are from two replicates of each condition at each time point. Only genera with a relative abundance > 1% in at least one sample are shown.
