# Supplementary material for: Exploring the roles of and interactions among microbes in dry co-digestion of food waste and pig manure using high-throughput 16S rRNA gene amplicon sequencing
Source: Biotechnol Biofuels. 2019 Jan 4;12:5. doi: 10.1186/s13068-018-1344-0 (PMC6318937; doi:10.1186/s13068-018-1344-0)
Supplement: Supplementary file 3 — Additional file 3: Table S1. Predicted anaerobic digestion-associated functions of bacterial taxa whose roles have not previously been reported in the literature. [file 13068_2018_1344_MOESM3_ESM.docx]

Table S1. Predicted anaerobic digestion-associated functions of bacterial taxa whose roles have not previously been reported in the literature

| Phylum | Class | Order | Family | Genus | Predicted function |
| --- | --- | --- | --- | --- | --- |
| *Firmicutes* | *Clostridia* | *Clostridiales* | *Caldicoprobacteraceae* | *Caldicoprobacter* | Syntrophic oxidation of acetate and butyrate |
|  |  | *MBA08* | *Unclassified* | *Unclassified* | Syntrophic oxidation of acetate and butyrate |
|  |  | *OPB54* | *Unclassified* | *Unclassified* | Syntrophic oxidation of acetate and butyrate |
|  |  | *BSA2B-08* | *Unclassified* | *Unclassified* | Syntrophic oxidation of acetate and butyrate |
|  |  | *SHA-98* | *Unclassified* | *Unclassified* | Syntrophic oxidation of acetate and butyrate |
|  |  |  | *D2* | *Unclassified* | Syntrophic oxidation of propionate |
| *Proteobacteria* | *Gammaproteobacteria* | *Pseudomonadales* | *Moraxellaceae* | *Acinetobacter* | Hydrolysis and fermentation, produce VFA, especially acetate and butyrate |
|  |  |  | *Pseudomonadaceae* | *Unclassified* | Hydrolysis and fermentation, produce VFA, especially acetate and butyrate |
| *Chloroflexi* | *Anaerolineae* | *Anaerolineales* | *Anaerolinaceae* | *SHD-231* | Hydrolysis and fermentation, produce VFA, especially acetate and butyrate |
| *Thermotogae* | *Thermotogae* | *Thermotogales* | *Thermotogaceae* | *S1* | Syntrophic oxidation of acetate and butyrate |
| *WWE1* | *Cloacamonae* | *Cloacamonales* | *Cloacamonaceae* | *W22* | Hydrolysis and fermentation, produce VFA, especially acetate and butyrate |
